# A Comparative Study of Autologous Stem Cell Transplantation (ASCT) Versus No ASCT in Newly Diagnosed Multiple Myeloma: A Single-Institution Experience From South India

**DOI:** 10.7759/cureus.105761

**Published:** 2026-03-24

**Authors:** Victor Mondal, Anand Praveen Kumar A, Abhinaya Sivaraman, Prabhat Ranjan, Steve Thomas

**Affiliations:** 1 Medical Oncology, Sri Ramachandra Institute of Higher Education and Research, Chennai, IND; 2 Medical Oncology, Stanley Medical College, Chennai, IND; 3 Medical School, Sri Ramachandra Institute of Higher Education and Research, Chennai, IND; 4 Haematology, Sri Ramachandra Institute of Higher Education and Research, Chennai, IND

**Keywords:** asct, multiple myeloma, retrospective, south india, survival

## Abstract

Background

Multiple myeloma (MM) is a clonal plasma cell malignancy with significant morbidity and mortality. Autologous stem cell transplantation (ASCT) has been a cornerstone in the treatment of newly diagnosed MM, but its role in the era of novel therapeutic agents remains under evaluation. Data from Indian populations are limited, particularly in the context of ASCT outcomes. This study aimed to compare the outcomes of ASCT versus no ASCT in newly diagnosed MM patients treated at a single institution in South India.

Methodology

A retrospective analysis was conducted among 59 patients diagnosed with MM between 2019 and 2023. In total, 11 patients underwent ASCT, while 48 did not. Patient demographics, treatment details, and outcomes were collected. Overall survival (OS) was estimated using the Kaplan-Meier method and compared using the log-rank test.

Results

The median OS for all patients was 35 months. Patients who underwent ASCT had a significantly longer median OS (61 months) compared to those who did not (not reached; p = 0.007). ASCT was associated with improved survival, particularly in patients with favorable prognostic factors such as younger age and lower International Staging System stage.

Conclusions

ASCT was associated with a significant survival benefit in patients with newly diagnosed MM in our cohort. Despite the emergence of novel agents, ASCT remains a valuable treatment option, particularly in selected patients with good performance status. Larger prospective studies are warranted to further define the role of ASCT in the era of modern therapies in the Indian context.

## Introduction

Multiple myeloma (MM) is a hematologic malignancy characterized by the clonal proliferation of malignant plasma cells within the bone marrow [1]. This proliferation leads to the production of monoclonal immunoglobulins (M-protein) and devastating end-organ damage, classically defined by the “CRAB” criteria: hypercalcemia, renal insufficiency, anemia, and bone lesions [2]. Globally, MM is the second most common blood cancer, and its incidence has been rising [3]. In India, MM accounts for approximately 1.2% of all newly diagnosed cancers, with a median age at diagnosis between 60 and 69 years, and a notable geographic concentration in the Southern and Northern zones [4].

For over two decades, the standard of care for transplant-eligible patients with newly diagnosed MM has been high-dose chemotherapy (HDT) followed by autologous stem cell transplantation (ASCT) [5]. This paradigm was established by landmark phase 3 trials in the 1990s and early 2000s. The Intergroupe Francophone du Myélome (IFM) 90 trial was pivotal, demonstrating that HDT-ASCT provided a significant improvement in both progression-free survival (PFS) and overall survival (OS) compared to conventional chemotherapy regimens [6]. This was subsequently confirmed by the MRC Myeloma VII trial in the United Kingdom [7].

The treatment landscape, however, has been revolutionized by the introduction of highly effective novel agents. Modern induction therapy, typically a triplet regimen combining a proteasome inhibitor such as bortezomib and an immunomodulatory drug (IMiD) such as lenalidomide with dexamethasone (known as VRd), can achieve deep and rapid responses before any consolidation is considered [8]. The profound efficacy of these novel agent-based regimens has triggered a critical debate: Is upfront ASCT still necessary?

This question has been addressed by two recent, large-scale phase 3 trials. The DETERMINATION trial (conducted in the United States) randomized patients to VRd induction followed by ASCT and lenalidomide (R) maintenance (VRd-ASCT-R) versus VRd induction alone followed by continuous R maintenance (VRd-R). After a median follow-up of 76 months, the trial showed a clear and significant PFS benefit for the ASCT arm (median PFS of 67.5 vs. 46.2 months). However, it reported no statistically significant difference in OS between the two groups [9]. The European FORTE trial compared a similar strategy using carfilzomib-based induction (KRd). It found that KRd followed by ASCT provided superior PFS compared to 12 cycles of KRd alone (no ASCT) [10].

Crucially, the non-ASCT arms in these trials relied on continuous, long-term maintenance therapy (typically lenalidomide) until disease progression. This strategy presents a profound challenge in resource-limited settings such as India. The “financial toxicity” of sustained novel agent therapy is a major barrier to care, making continuous maintenance unfeasible for a large proportion of patients [11]. Real-world data from India already highlights significant deviations from Western protocols, including long delays from diagnosis to transplant (median >12 months) due to financial constraints and institutional capacity [12].

Therefore, the “no ASCT” arm in a real-world Indian cohort is not a true comparator to the “VRd-R” arm of the DETERMINATION trial. It is a heterogeneous group consisting of transplant-ineligible patients and eligible patients who may have opted out due to cost or comorbidities, and who likely receive varied and often intermittent follow-up therapy. Given this “treatment access gap,” there is a critical need for regional, real-world data. Therefore, the primary objective of this study was to evaluate and compare OS outcomes between patients with newly diagnosed MM who underwent ASCT and those who did not in a retrospective single-center cohort from a resource-limited setting in South India.

## Materials and methods

Study design and patient selection

This retrospective, single-center study included all patients diagnosed with MM and registered in our Medical Oncology department from January 2019 to December 2023.

Transplant eligibility criteria

Transplant eligibility was assessed at diagnosis based on institutional protocols, considering age (typically ≤70 years), Eastern Cooperative Oncology Group (ECOG) performance status (0-2), adequate organ function (cardiac, pulmonary, hepatic, and renal), and the absence of prohibitive comorbidities. Patient choice and socioeconomic factors, including financial toxicity, were also significant determinants in the final treatment decision.

Pre-transplant evaluation

All patients underwent a standard diagnostic workup, including medical history, physical examination, and staging via the International Staging System (ISS). Baseline laboratory tests included a complete blood count, comprehensive metabolic panel, serum beta-2 microglobulin, lactate dehydrogenase, serum protein electrophoresis (SPEP), immunofixation, quantitative immunoglobulins, and serum free light-chain (FLC) assay. A 24-hour urine collection was used for urine SPEP. Bone marrow aspiration and biopsy (with cytogenetics/fluorescence in situ hybridization (FISH)) and a skeletal survey were performed.

Induction and maintenance protocols

Patients received induction therapy per institutional guidelines, adapted for age, frailty, and renal function. The most common triplet regimens were VRd (bortezomib, lenalidomide, dexamethasone) and VCD (bortezomib, cyclophosphamide, dexamethasone). Bortezomib was typically administered subcutaneously at 1.3 mg/m² (weekly or bi-weekly), and dexamethasone at 20-40 mg weekly. Lenalidomide dosing (typically 10-25 mg on days 1-21 of a 28-day cycle) was adjusted for baseline creatinine clearance. Following induction (and ASCT, if applicable), maintenance therapy with single-agent lenalidomide (10 mg daily) was universally recommended. However, due to the resource constraints highlighted in this study, actual adherence to continuous maintenance was highly variable. Patients in both arms frequently experienced treatment interruptions, dose reductions, or premature cessation of maintenance primarily due to financial toxicity rather than medical intolerance.

Stem cell collection and transplantation

Peripheral blood stem cells were mobilized using granulocyte colony-stimulating factor (G-CSF) with or without chemotherapy and collected via leukapheresis after induction therapy. Cells were cryopreserved or stored at 4°C for fresh infusion, occurring 24 hours after completion of conditioning.

Conditioning regimen and supportive care

The standard conditioning regimen was high-dose melphalan (200 mg/m² or dose-reduced to 140 mg/m² for renal impairment). Some patients received bortezomib in addition to melphalan. Supportive care included G-CSF post-transfusion and antimicrobial prophylaxis (fluoroquinolone, fluconazole, and acyclovir).

Follow-up and outcome measures

Patients in both the ASCT and non-ASCT cohorts were monitored systematically in the outpatient clinic. The standard follow-up schedule included monthly visits for the first year post-induction/transplant, and every three months thereafter, or more frequently if clinically indicated. Routine follow-up assessments included a complete blood count, comprehensive metabolic panel, SPEP with immunofixation, and serum FLC assays. Bone marrow evaluation and advanced imaging (such as PET-CT or whole-body MRI) were reserved for cases of suspected biochemical or clinical relapse. Response and disease progression were defined strictly according to the International Myeloma Working Group (IMWG) 2016 criteria [13]. The primary endpoints were PFS, defined as the time from diagnosis to the first documented disease progression or death from any cause, and OS, defined as the time from diagnosis to death from any cause.

Statistical analysis

Patient characteristics were summarized using descriptive statistics. OS and PFS were estimated using the Kaplan-Meier method. The log-rank test was used to compare survival curves between the ASCT and non-ASCT groups. A p-value <0.05 was considered statistically significant. All analyses were performed using SPSS Statistics version 26 (IBM Corp., Armonk, NY, USA).

## Results

A total of 59 patients with newly diagnosed MM registered between January 2019 and December 2023 were included in this retrospective analysis. The median follow-up time for the entire cohort was 38.4 months (95% confidence interval (CI) = 31.2-45.6 months). Baseline demographic and disease characteristics for the entire cohort, stratified by treatment group (ASCT vs. non-ASCT), are summarized in Table 1.

**Table 1:** Patient characteristics. The table summarizes the baseline characteristics of the study cohort. n: number of patients; %: percentage. Test statistics represent the Mann-Whitney U value for continuous variables (age) and Pearson’s chi-square value for categorical variables. ASCT = autologous stem cell transplantation; ECOG PS = Eastern Cooperative Oncology Group performance status; ISS = International Staging System; eGFR = estimated glomerular filtration rate

Characteristic	Total cohort (N = 59)	ASCT group (n = 11)	Non-ASCT group (n = 48)	Test statistic*	P-value
Median age, years (range)	61 (34–79)	54 (34–65)	63 (40–79)	U = 125.0	0.009
Sex, n (%)				Chi-square = 0.15	0.71
Male	40 (67.8%)	8 (72.7%)	32 (66.7%)		
Female	19 (32.2%)	3 (27.3%)	16 (33.3%)		
ECOG PS, n (%)				Chi-square = 2.05	0.15
0–1	43 (72.9%)	10 (90.9%)	33 (68.8%)		
≥ 2	16 (27.1%)	1 (9.1%)	15 (31.2%)		
ISS stage, n (%)				Chi-square = 2.44	0.28
I	12 (20.3%)	4 (36.4%)	8 (16.7%)		
II	21 (35.6%)	4 (36.4%)	17 (35.4%)		
III	26 (44.1%)	3 (27.2%)	23 (47.9%)		
Renal impairment (eGFR <50), n (%)	23 (39.0%)	2 (18.2%)	21 (43.8%)	Chi-square = 2.45	0.08
High-risk cytogenetics, n (%)	9 (22.0%)	3 (27.3%)	6 (20.0%)	Chi-square = 0.22	0.78
Triplet induction regimen, n (%)	46 (78.0%)	11 (100.0%)	35 (72.9%)	Chi-square = 4.02	0.04

Demographics

The median age for the entire cohort was 61 years (range = 34-79 years), with 40 (67.8%) being male. As expected, patients in the ASCT group were significantly younger than those in the non-ASCT group (median age = 54 years vs. 63 years, p = 0.009).

Performance status

A difference was observed in baseline ECOG performance status (PS). Notably, 10 (90.9%) patients in the ASCT group had a good PS of 0-1, whereas 15 (31.2%) patients in the non-ASCT group had a poor PS of 2 or more at presentation.

Disease burden and stage

At diagnosis, 23 (39.0%) of all patients presented with renal impairment (defined as estimated glomerular filtration rate <50 mL/minute). Disease stage, classified by the ISS, was distributed as follows for the entire cohort: Stage I, 12 (20.3%); Stage II, 21 (35.6%); and Stage III, 26 (44.1%). The non-ASCT group had a notably higher proportion of patients with ISS Stage III disease compared to the ASCT group (47.9% vs. 27.2%).

Cytogenetic risk

Cytogenetic data via FISH were available for 41 (69.5%) patients. High-risk cytogenetics, defined by the presence of del(17p), t(4;14), or t(14;16), was identified in nine (22.0%) patients. The distribution of high-risk disease was comparable between the two groups (27.3% in ASCT vs. 20.0% in non-ASCT, p = 0.78).

Induction therapy

The most common induction regimen was a bortezomib-based triplet (VRd or VCD), received by 46 (78.0%) of all patients. All 11 (100.0%) patients in the ASCT group received a triplet induction, compared to 35 (72.9%) in the non-ASCT group. Before ASCT, eight (72.7%) of the transplant patients had achieved at least a very good partial response.

Survival outcomes

**Figure 1:**
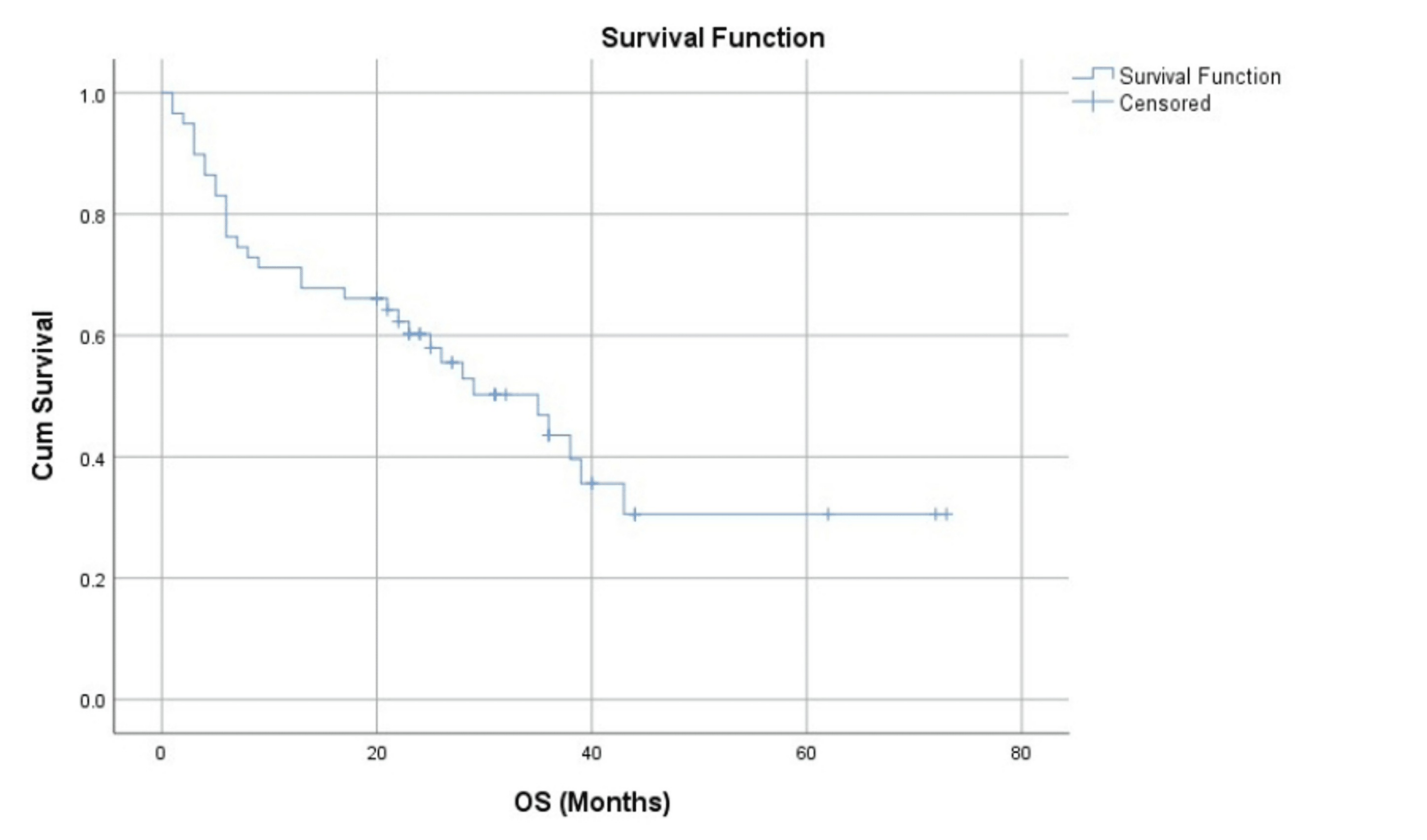
Overall survival. Kaplan-Meier survival curve illustrating the OS for the entire cohort of newly diagnosed multiple myeloma patients (N = 59). OS = overall survival

**Figure 2:**
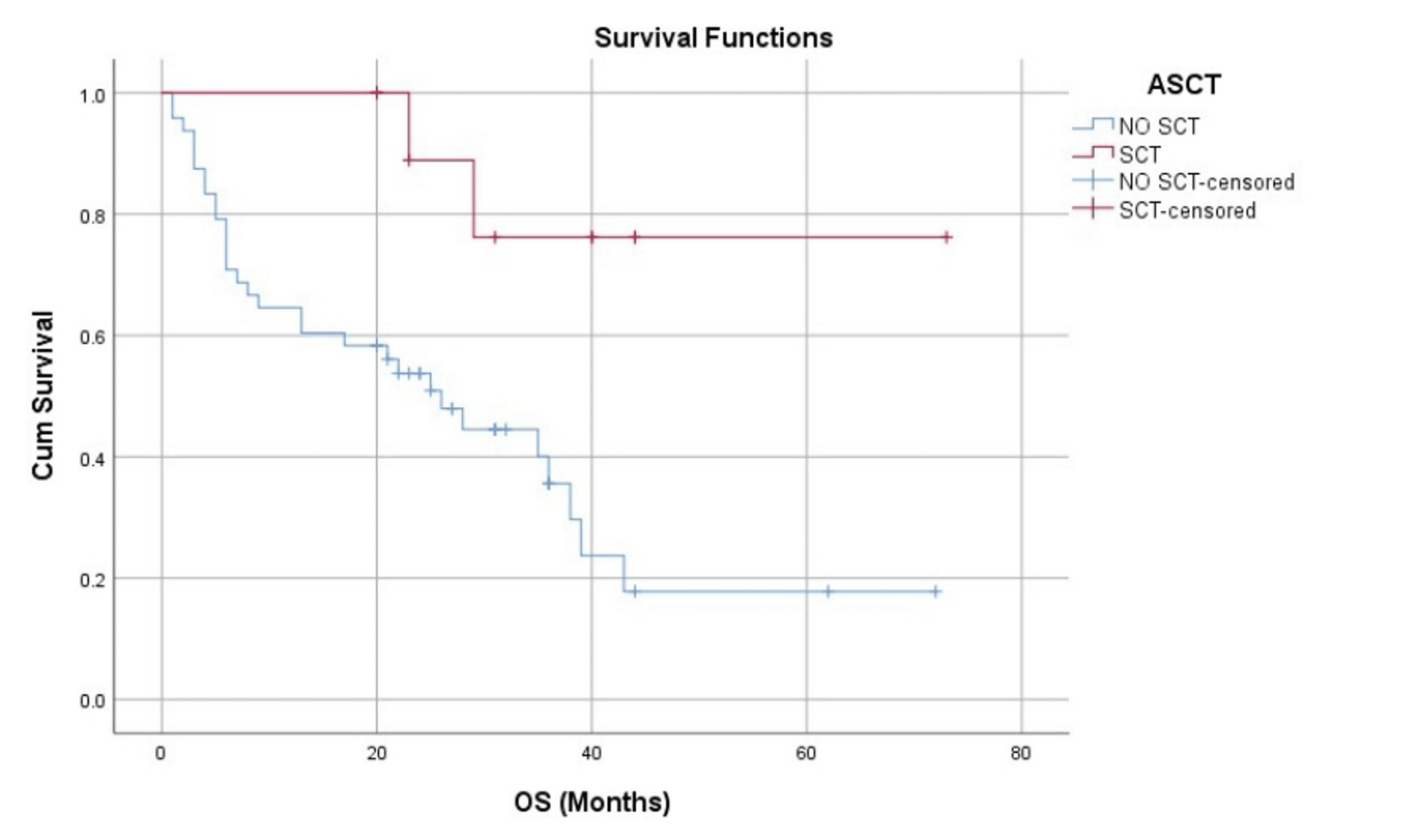
Overall survival ASCT versus no ASCT. Kaplan-Meier survival curve comparing the OS between the ASCT group and the non-ASCT group, demonstrating a statistically significant survival benefit in the transplant arm (log-rank p = 0.007). ASCT = autologous stem cell transplantation; OS = overall survival

The primary endpoint of OS demonstrated a significant difference between the two treatment arms (Figure 1). For the entire cohort, the median OS was 35 months (95% CI = 22-47 months). A clear survival advantage was seen in the transplant arm. The median OS for the ASCT group was not reached at the time of data cutoff, as more than 50% of the patients remained alive. In contrast, the median OS for the non-ASCT group was 35 months (95% CI = 20-49 months). The Kaplan-Meier survival curves for the two groups separated early and continued to diverge throughout the follow-up period (Figure 2). Importantly, despite the small number of patients in the ASCT cohort (n = 11), the divergence of these curves is so marked that it suggests a very strong biological and clinical effect, driving a highly statistically significant durable survival advantage for the ASCT arm (log-rank p = 0.007).

## Discussion

Our single-institution, retrospective analysis of 59 patients with newly diagnosed MM in South India reveals a stark and statistically significant OS benefit for patients who received upfront ASCT. The median OS for the ASCT group was not reached, whereas the non-ASCT group had a median OS of 35 months (log-rank p = 0.007). This finding, while preliminary, is critical in the context of the ongoing global debate regarding the role of upfront ASCT in the era of novel therapies. While we acknowledge the limitation of the small sample size in the transplant cohort, the early and striking separation of the survival curves underscores a potent clinical benefit that transcends the low patient numbers.

This result appears to stand in direct contrast to the headline findings of two of the most important recent phase 3 trials: the US-based DETERMINATION trial and the European FORTE trial [9,10]. Both studies compared modern triplet induction (VRd or KRd) followed by ASCT against a strategy of continuous triplet therapy without ASCT, with both arms receiving lenalidomide maintenance. While both trials confirmed that the ASCT arm achieved a superior and significantly longer PFS, neither has yet demonstrated a statistically significant OS benefit after more than six years of follow-up [9].

The apparent paradox between our findings (a clear OS benefit) and those of major Western trials (a PFS-only benefit) illuminates a fundamental truth about treatment in a resource-constrained environment. A primary confounder in interpreting these results is selection bias. The non-ASCT group (81.4% of our cohort) is an inherently heterogeneous and poor-prognosis population. It includes all patients deemed transplant-ineligible due to advanced age, poor performance status, or significant comorbidities (e.g., renal failure). It also includes eligible patients who may have opted out due to socioeconomic factors or fear of toxicity. This group’s poor median OS of 35 months is therefore a reflection of both their underlying biology and their inability to access high-intensity treatment, not just the omission of ASCT.

In our South Indian cohort, the real-world comparator differs significantly from clinical trial comparators. It is highly improbable that the patients in the non-ASCT group received this clinical trial standard. Due to significant financial toxicity and access barriers, continuous lenalidomide maintenance is not a feasible long-term strategy for the majority of patients in this setting [11,14]. Therefore, the non-ASCT arm in our study is not “VRd + continuous maintenance”; it is more accurately “Induction ± intermittent/no maintenance.” In the DETERMINATION and FORTE trials, the no-ASCT arm received highly effective triplet induction followed by continuous lenalidomide maintenance until disease progression [9,10]. This distinction is fundamental to the integrity of our comparative analysis: the observed OS benefit of ASCT in our cohort reflects its vital role as a highly potent consolidation strategy when sustained, optimal maintenance therapy is financially or logistically inaccessible. The demonstrated efficacy of lenalidomide maintenance, proven in landmark trials such as CALGB 100104 (McCarthy et al.) [15] and IFM 2005-02 (Attal et al.) [16] to significantly improve PFS and OS, is the essential component that makes the “no ASCT” arm a viable strategy.

Viewed through this lens, our study is comparing induction plus ASCT against induction plus limited follow-up therapy. In this specific and common real-world scenario, the high-dose melphalan and ASCT act as a powerful, time-limited consolidation that provides a depth and durability of response that induction alone simply cannot match. Where sustained maintenance is not guaranteed, the “one-shot” benefit of ASCT becomes paramount.

The central role of response depth also provides context to our findings. Modern myeloma care aims for deep, undetectable responses, measured as minimal residual disease (MRD) negativity, which is the single most important prognostic factor for long-term survival [17,18]. ASCT remains one of the most effective and reliable methods for achieving MRD negativity. Data from the Myeloma XI trial clearly showed that achieving an MRD-negative state post-ASCT was a powerful predictor of improved PFS and OS [19]. Furthermore, recent data suggest that even for excellent responders who achieve MRD negativity after induction, proceeding with ASCT provides additional benefit. A 2023 study by Liu et al. demonstrated that these patients still had significantly prolonged PFS and OS if they received ASCT consolidation, suggesting ASCT provides a level of cytoreduction that induction alone cannot [20]. In our cohort, it is this consolidation of response that likely drives the OS benefit, as patients in the non-ASCT arm who relapse are less likely to have durable remissions.

Reconciling the PFS and OS outcomes requires analyzing post-relapse care. The primary reason the clear PFS benefit in the DETERMINATION trial did not translate to an OS benefit is that patients who relapsed in the no-ASCT arm had access to numerous, highly effective salvage therapies, including next-generation IMiDs, monoclonal antibodies, and delayed ASCT [9]. Effective salvage rescued the OS in the non-transplant arm. In a resource-limited setting, access to this sequence of effective salvage therapies is severely restricted. A first relapse is often a catastrophic and life-limiting event. Therefore, in our context, the initial PFS benefit is critical and directly translates to an OS benefit, which is precisely what our data shows. The goal must be to achieve the deepest and longest first remission possible, and ASCT remains our most potent tool to do so.

This study is constrained by several significant limitations inherent to its retrospective, single-center design. Foremost is the severe selection bias and cohort heterogeneity. Unlike controlled clinical trials (such as the DETERMINATION trial), where the non-transplant arm receives continuous, standardized therapy, our non-ASCT group represents a true real-world population. This group includes patients who inherently had greater clinical frailty (older age, worse ECOG performance status) and higher disease burden (ISS Stage III), but also those who opted out of ASCT due to socioeconomic barriers. Consequently, their subsequent maintenance therapy was likely intermittent or nonexistent due to financial toxicity. Therefore, while our findings demonstrate a strong association between ASCT and improved survival, the observed benefit is undeniably influenced by both the efficacy of the transplantation and the better baseline prognosis of the patients who were able to access it. Furthermore, the small sample size, specifically the low number of patients (n = 11) in the ASCT arm, precluded the use of advanced statistical models, such as propensity score matching, to accurately isolate the transplant-independent effect.

Data maturity and completeness present additional, critical limitations. Although the median follow-up was 38.4 months, several patients in the ASCT arm have not yet experienced progression events, which limits the maturity of the long-term survival data. Crucially, MRD data were unavailable for this cohort. Because MRD status is currently the strongest predictor of survival in myeloma, the lack of this metric prevents us from definitively confirming whether the ASCT advantage in our cohort is strictly due to a greater depth of biological response or other supporting clinical factors. Finally, the absence of FISH data in approximately 30% of the cohort limits our ability to fully rule out underlying cytogenetic risk imbalances between the two groups. Despite these limitations, we present these findings as vital, real-world, hypothesis-generating evidence underscoring the critical role of upfront consolidation when sustained novel-agent therapy is inaccessible.

## Conclusions

In this retrospective real-world cohort, ASCT was associated with improved OS in newly diagnosed MM patients. However, as noted, this finding may be influenced by selection bias and differences in baseline characteristics. We present these findings as real-world, hypothesis-generating evidence that in resource-limited settings where access to continuous maintenance and subsequent salvage therapies is restricted, upfront ASCT plays a critical role in consolidating disease and improving outcomes. Future efforts should focus on developing prospective, multi-center Indian myeloma registries to track real-world outcomes and establish evidence-based, resource-stratified treatment guidelines.
